# Comparisons of different electrical stimulation modalities for treating visceral pain in a rodent model of irritable bowel syndrome

**DOI:** 10.1186/s42234-024-00158-1

**Published:** 2024-11-11

**Authors:** Md Jahangir Alam, Tingting Zhao, John W. Wiley, Jiande D. Z. Chen

**Affiliations:** Division of Gastroenterology and Hepatology, Department of Internal Medicine, University of Michigan, Ann Arbor, MI 48109 USA

**Keywords:** Electroacupuncture, Transcutaneous electrical acustimulation, Visceral pain, Irritable bowel syndrome

## Abstract

The purpose of this study was to investigate the effects of different electrical stimulation methods (bilateral electroacupuncture (BEA), unilateral EA (UEA), transcutaneous electrical acustimulation (TEA, stimulation via surface electrodes placed at acupoints), and sacral nerve stimulation (SNS)) on visceral pain in a rodent model of irritable bowel syndrome (IBS). Ten-day-old male and female pups were treated with 0.2 ml of 0.5% acetic acid (AA) solution. Visceral sensitivity was assessed using an electromyogram (EMG) in response to graded colorectal distension. In the first experiment, bilateral EA at ST36 acupoint was performed with different parameters in male rats to determine the best stimulation parameters. In the second experiment, male rats were randomly assigned into the Sham, BEA, UEA, TEA, and SNS groups to determine the best stimulation method. Lastly, the AA-treated female rats were randomly assigned into the BEA and sham groups to investigate a potential treatment difference between the sexes. Two distinct sets of stimulation parameters were used: Set 1 (100 Hz, 0.5 ms pulse width, 0.1 s ON, 0.4 s OFF, 0.4–3.0 mA current) and Set 2 (25 Hz, 0.5 ms pulse width, 2 s ON, 3 s OFF, 0.4–3.0 mA current).

**Results **(1) The parameter set of 100Hz was found to be most effective in reducing visceral pain. (2) Both acute UEA and TEA effectively relieved visceral pain, whereas acute SNS did not exhibit such an effect. (3) Acute BEA improved visceral pain in both male and female rats.

**Conclusions** These findings suggest that transcutaneous ST36 stimulation is as effective as direct ST36 stimulation and unilateral ST36 stimulation is comparable to bilateral stimulation. Development of a novel therapy using unilateral transcutaneous ST36 stimulation is warranted.

## Introduction

Irritable bowel syndrome (IBS) is one of the most common gastrointestinal (GI) disorders, which is characterized by the occurrence of chronic and recurrent abdominal pain, bloating, distention, and changes in bowel habits. IBS affects between 5-10% of the global population (Chey et al. 2015; Mayer et al. 2023), and women are at higher risk for IBS than men. The etiology of pain in IBS is not well understood. However, clinical and animal studies suggest that sensitization of visceral afferents, spinal dorsal horns, and dysfunction of the descending modulatory systems may have an important role. The brain receives nociceptive pain signals from the visceral organs, including the GI tract, and inputs from these overly sensitive nerves are responsible for pain perception. This bidirectional interaction between the brain and gut is essential for maintaining GI homeostasis and higher cognitive functions (Alam and Chen 2023a; Alam and Chen 2023b; Deiteren et al. 2016). Visceral hypersensitivity, characterized by hypersensitivity to a stimulus, is another crucial feature of IBS and is thought to underlie abdominal pain in patients with IBS. Several mechanisms may contribute to this feature, including mast cell activation, increased mucosal permeability, sensitization of visceral afferents, and dietary habits. Psychiatric comorbidities such as depression and anxiety are prevalent in IBS patients and correlate with enhanced visceral pain perception, which may play a role in the pathogenesis of IBS (Alam and Chen 2023a; Alam and Chen 2023b; Deiteren et al. 2016).

The pathogenesis of IBS is complicated and multifactorial; therefore, treating pain in IBS is challenging. Standard treatment methods include anticholinergic agents, antidepressants, such as tricyclic antidepressants (TCAs), serotonin reuptake inhibitors (SSRIs), and monoamine uptake inhibitors. Anticholinergic agents such as hyoscyamine and dicyclomine reduce abdominal pain and discomfort by reducing spasms or contractions in the intestine. Antidepressants decrease pain perception by regulating nerve signaling and can potentially increase or decrease GI function. These drugs have therapeutic effects on mood, sleep, and associated psychological disturbances (Bahar et al. 2008; Chen et al. 2017; Crowell et al. 2004; Grover and Drossman 2008). SSRIs improve overall well-being, reduce anxiety associated with IBS, and enhance the analgesic properties of TCAs, suggesting that SSRIs may decrease pain in IBS patients. Serotonin receptors (e.g., Tegaserod and Alosetron) increase gut movement and intestinal secretions by working on the nerves and GI smooth muscles. These agents improve pain and bloating in IBS patients (Camilleri et al. 2001; Camilleri et al. 2000; Ford et al. 2009; Shah et al. 2021). Although these drugs improve pain and overall symptoms in IBS, unsatisfactory side effects, such as headache, dizziness, dry mouth, insomnia, cardiovascular disorders, and ischemic colitis, exist. Due to the disease's heterogeneous nature, treatment options are minimal and often controversial; therefore, alternative treatment methods, such as electrical neuromodulation, could be beneficial.

Neuromodulation is an emerging field in medical sciences that modulates or changes the functioning of the central, peripheral, or autonomic nervous system. It acts directly or indirectly on nerves and alternates or modulates nerve activity using electrical, chemical, and mechanical interventions (Chen et al. 2022). Neuromodulation can be invasive and noninvasive. Invasive neuromodulation requires a surgical procedure to implant stimulating electrodes. Sacral nerve stimulation (SNS) is one of the most common invasive neuromodulation methods. It is FDA-approved for treating overactive bladder and fecal incontinence; its potential for treating pain in IBS has also been explored (Fassov et al. 2019). Noninvasive neuromodulation typically involves transcutaneous electrical stimulation that can penetrate the skin to stimulate nerves. Some of these methods include electroconvulsive therapy (ECT), transcranial electrical stimulation (TES), transcranial direct current stimulation (tDCS), electroacupuncture (EA), transcutaneous auricular vagal nerve stimulation (taVNS), transcutaneous electrical acustimulation (TEA) or transcutaneous electrical acupoint stimulation (TEAS), and transcutaneous tibial nerve stimulation (tTNS) (Yin and Chen 2023).

Preclinical and clinical studies have suggested that EA and TEA could be used for managing pain in IBS (Nojkov et al. 2024). TEA replaces traditional acupuncture needles with surface electrodes, providing a nonpharmaceutical alternative for pain management. This technique involves the application of low-intensity electrical stimulation, with specific parameters tailored for the treatment of abdominal pain, via surface electrodes positioned at targeted acupuncture points near peripheral nerves. For instance, TEA at acupoint ST36—adjacent to the peroneal, sciatic, and tibial nerves—and at PC6, located near the median nerve, has been shown to improve bowel movements, alleviate abdominal pain, and enhance colon transit and rectal sensation through autonomic mechanisms in patients with constipation-dominant IBS (Huang et al. 2022). EA at the ST36 acupoint with a frequency of 100 Hz effectively enhanced rectal compliance and alleviated visceral hypersensitivity in rats with intestinal inflammation induced by 5% dextran sulfate sodium (DSS) (Chen et al. 2021a).

Preliminary clinical studies from our group with TEA at bilateral acupuncture points, ST36, have shown an analgesic effect in patients with IBS (Huang et al. 2022; Hu et al. 2022). However, bilateral stimulation limits the mobility of patients. Therefore, it is essential to determine whether unilateral stimulation on one leg is equally effective as this would allow patients to resume daily activity during the treatment. In addition, it is unknown whether the noninvasive ST36 simulation method via surface electrodes is as potent as ST36 stimulation via inserted needles or direct electrical stimulation of the sacral nerve that innervates the colon (SNS), which has been clinically used for treating various pelvic floor disorders (Fassov et al. 2019; Siegel et al. 2001).

Accordingly, this study aimed to investigate the effects of different stimulation modalities on visceral hypersensitivity in a rodent model of IBS by comparing different stimulation parameters, bilateral vs. unilateral stimulation, EA via needles vs. TEA via surface electrodes, and EA vs. SNS. Furthermore, we investigated potential treatment differences between males and females.

## Materials and methods

### Animals

Forty-six Sprague-Dawley rat pups (28 males and 18 females) were purchased from Charles River Laboratories in Kingston, NY, USA, at the age of nine days. On postnatal day 10 (P10), the rat pups received a manual intracolonic injection of 0.2 mL of 0.5% acetic acid (AA) into the distal colon, specifically 2 cm from the anus (Al-Chaer et al. 2000). Food and water were provided ad libitum, and all animals were maintained at room temperature under a 12:12-hour light/dark cycle.

### Surgical procedures

Electrode implantation surgery was performed during 8–9 weeks of age for four different purposes: Bilateral electroacupuncture (BEA), Unilateral electroacupuncture (UEA), Sacral nerve stimulation (SNS), and recording abdominal electromyography (EMG). Rats were anesthetized with 2% isoflurane (Piramal Critical Care Inc., Bethlehem, PA, USA) with a 2 liter/min oxygen flow. The body temperature was maintained at 37°C during the surgery, and an ophthalmic ointment was applied to the eyes to prevent dryness.

#### EMG electrode implantation

A pair of stainless-steel wires (Cardiac pacing wires, A&E Medical, Farmingdale, NJ, USA) was inserted into the abdominal muscle to record the EMG response to colorectal distention (CRD) (Jiang et al. 2019; Jin et al. 2021).

#### ST36 electrode implantation

A pair of the same wires was inserted bilaterally into acupoints ST36, located 5 mm below the head of the fibula, under the knee joint, and 2 mm lateral to the anterior tubercle of the tibia in rats. The electrode wires were inserted bilaterally at a depth of 5 mm into the muscles at ST36 and secured with sutures (Jin et al. 2019). For unilateral ST36 stimulation, another electrode was placed 5 mm below the ST36 acupoint on either the left or right leg.

#### SNS electrode implantation

A dorsal midline incision was made to expose the right sacral nerve. One pair of electrodes (Streamline 6494F, Medtronic, Minneapolis, MN, USA) were placed around the right sacral nerve (S1) behind the sacral foramen and fixed by a surgical knot (oval cathode 2–3 mm in length in each electrode). To isolate the exposed wires from the adjacent tissues, we used Kwik-Sil (World Precision Instruments, Sarasota, FL, USA) on the wires (Jiang et al. 2019; Tu et al. 2020a).

**Fig 1:**
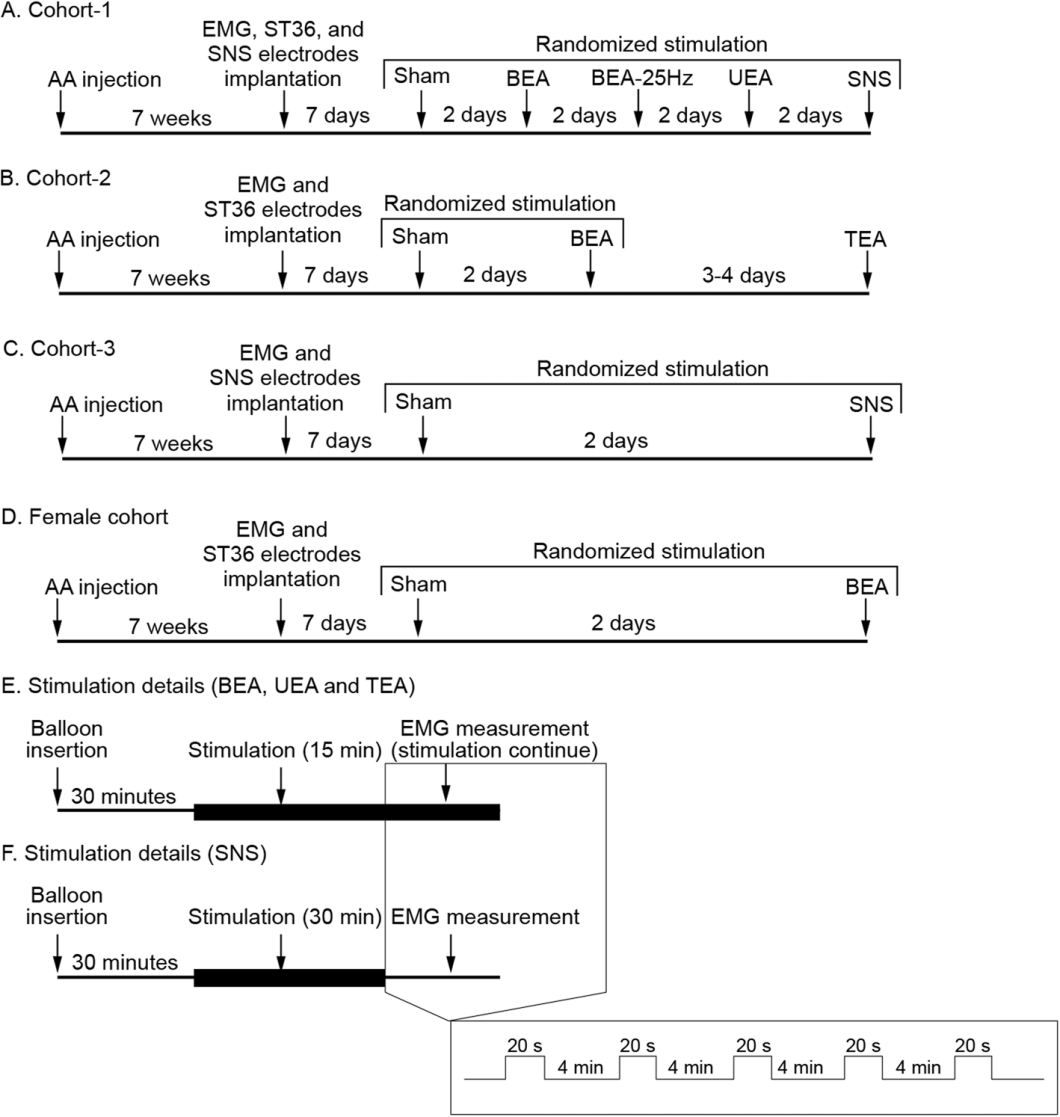
Experimental timeline

All electrode connecting wires were tunneled subcutaneously and brought out at the back of the neck. Post-surgery, Carprofen (5 mg/kg) and Enrofloxacin (5 mg/kg) were administered for two days to control infection and postoperative pain, respectively. The rats were allowed to recover for seven days before the experiment. All male and female rats received electrode implantation in the abdomen. Among the 28 male rats, 11 received electrode implantation in the S1 and ST36 locations (cohort-1, Fig 1A), 9 in the ST36 location (cohort-2, Fig 1B), and 8 in the S1 location (cohort-3, Fig 1C), while female rats received electrode implantation in the ST36 location (Female cohort, Fig 1D).

Rats received stimulation in a randomized order, with each lasting approximately one hour. The EMG was recorded following each stimulation session in response to graded colorectal distension (CRD). Following balloon insertion, the rats were allowed to rest in the restrainer for 30 minutes before the stimulation session began. In the BEA, UEA, and TEA groups, EMG responses were measured after 15 minutes of stimulation, and stimulation continued throughout the EMG measurements, except during balloon inflation (Fig. 1E). In the SNS group, EMG responses were measured after 30 minutes of stimulation (Fig. 1F). Black vertical bar represents the stimulation period.

### Experimental design

In this study, we used forty-six (*n* = 46) neonatally AA-treated rats. In the first cohort, rats were randomized for Sham (stimulation output set at 0mA), BEA, UEA, BEA-25Hz, and SNS stimulation (Fig 1A). In the second cohort, rats underwent Sham and BEA in randomized order (Fig 1B). Three to four days after the last stimulation, rats were subjected to TEA (Fig 1B). The third cohort of rats received Sham and SNS stimulation in a randomized order (Fig 1C). Among the 18 male rats, 13 received Sham or BEA in randomized order (Fig 1D).

Thirty minutes after inserting a balloon under isoflurane anesthesia (see Visceromotor reflex (VMR) response to CRD section below for details), electrical stimulation was delivered via the stimulating wires using a digital stimulator (World Precision Instrument, Sarasota, FL, USA). After 15 minutes of stimulation (BEA, UEA, or TEA), EMG responses were measured with graded colorectal distension (CRD). The stimulation was continuous except during the EMG measurement (Fig. 1E). However, the SNS group received 30 minutes of stimulation and no stimulation during the EMG measurement (Fig. 1F).

#### BEA

BEA was performed using two different sets of parameters: Set 1: 0.1s on, 0.4s off, 100Hz, and 0.5ms pulse width; this parameter was previously used for relieving visceral pain (Chen et al. 2021a; Sun et al. 2014) and Set 2: 2s on, 3s off, 25Hz, 0.5ms; this parameter was shown to accelerate gastric motility in previous studies (Song et al. 2013; Yin et al. 2010). In the case of both stimulation parameters, the amplitude was set at the motor threshold (MT) to evoke muscle contractions surrounding ST36 acupoints (0.4~3.0 mA).

#### UEA

UEA was performed using stimulation parameter set 1. Stimulation was performed using one electrode inserted at ST36 and another electrode inserted at 5 mm below ST36. The stimulation amplitude was determined to be the minimum current required to induce muscle contraction surrounding ST36 acupoints.

#### SNS

SNS was performed using parameter set 1 and the amplitude was set at 80% of the MT (0.4~2.0 mA). The MT is defined as the stimulation amplitude required to elicit the first observable motor response of the rodent tail.

#### TEA

TEA was achieved bilaterally using surface patch electrodes, and a watch-size digital stimulator (SNM-FDC01, Transtimulation Research Inc, OK, USA) was used to deliver electrical stimulation. Before the attachment of electrodes, the hair and area of ST36 were shaved and cleaned using alcohol. Then, a conducting gel was applied to reduce impedance, and one electrode was placed over each ST36 point and fixed with tape. Stimulation parameter set 1 was used and the amplitude was set at a level that induced contractions of muscle surrounding ST36 (0.3-5.0 mA)

### Visceromotor reflex (VMR) response to CRD

We employed a previously established method (Al-Chaer et al. 2000; Chen et al. 2021b) to assess the visceromotor reflex in response to CRD. Under mild sedation with 1-2% isoflurane, a flexible balloon (5 cm) constructed from a surgical glove finger attached to a Tygon tube was inserted into the descending colon and rectum 8cm from the anal verge and held in place by taping the tube to the tail. The rat was placed in a transparent restrainer and allowed to adapt for 30 min before the test. CRD was performed by rapidly inflating the balloon to predefined constant pressures of 10, 20, 40, 60, and 80 mmHg for a 20-s period, each followed by a 4-minute rest at a pressure of 0 (Fig. 1E, F). After 4 minutes of rest, the whole process was repeated one more time. The EMG response was recorded continuously during the experiment using a Biopac system EMG 100C (Biopac Systems Inc., Goleta, CA, USA). The EMG signal was amplified from 1Hz to 5000 Hz and digitized using the Acknowledge (Biopac Systems, Inc.). The area under the curve (AUC) of the EMG signal during each 20s distention period was calculated using an in-house written computer program (Jiang et al. 2019). The net EMG value for each distension, representing the strength of visceromotor reflexes, was calculated by subtracting the baseline value derived from the AUC for the 20s pre-distention period.

#### Data exclusion

During our study, we encountered specific instances where data had to be excluded to uphold the integrity and reliability of our analysis. These exclusions were carried out in accordance with standard scientific practices and guidelines. One male rat was removed from the dataset due to an outlier value observed during the Sham EA stimulation, and subsequently, the rat died. Five female rats were not subjected to acute BEA stimulation. As a result, they were not included in the subsequent analysis.

### Statistical analysis

All data are presented as mean ± SEM. Statistical analyses were performed using Prism version 10 software (GraphPad). Multiple group comparisons were assessed using one-way, two-way, or repeated-measures ANOVA, followed by the appropriate post hoc test when significant main effects or interactions were detected. The null hypothesis was rejected at the *p* < 0.05 level.

## Results

### Effects of different electrical stimulation methods on visceral pain in AA-treated male rats

#### Acute BEA improved visceral pain

**Fig 2:**
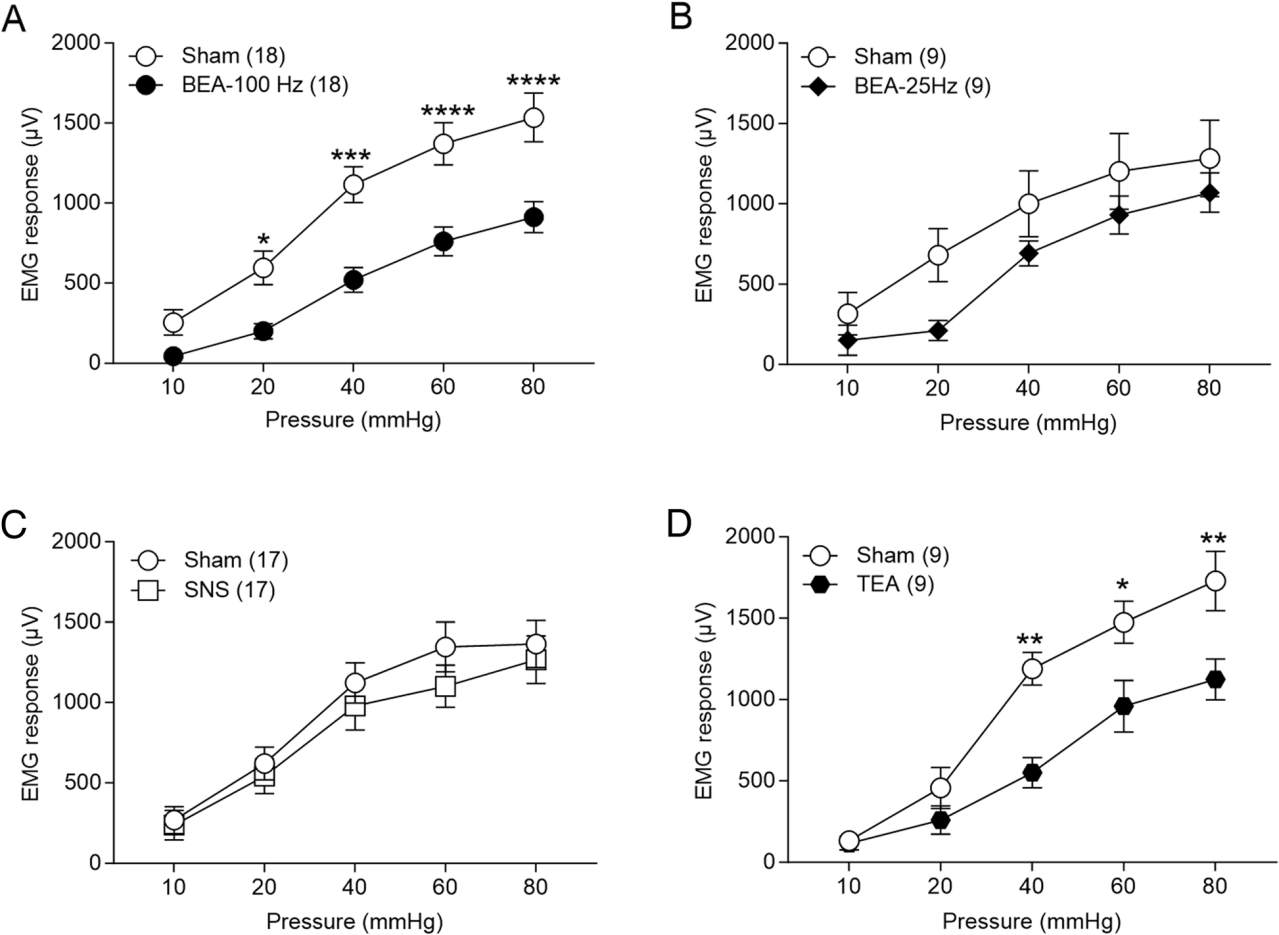
Ameliorating effect of acute BEA in AA-treated male rats. BEA-100Hz, but not BEA-25Hz, reduced pain intensity in AA-treated male rats. **A** Acute BEA-100Hz decreased EMG responses compared to the Sham group (Two-way repeated measures ANOVA, *F* (4, 136) = 4.42, *p* = 0.002). **B** BEA-25Hz had no effect on pain sensitivity in AA-treated male rats (Two-way repeated measures ANOVA, *F* (4, 64) = 1.18,* p* = 0.326). **C** Acute SNS did not improve visceral pain in AA-treated male rats. The SNS group had comparable EMG responses to the Sham group (Two-way repeated measures ANOVA, *F* (4, 128) = 0.72,* p* = 0.578). **D** Acute TEA improved visceral pain in AA-treated male rats. TEA, similar to BEA-100Hz, decreased EMG responses compared to the Sham group and improved visceral pain in AA-treated male rats (Two-way repeated measures ANOVA*, F* (4, 64) = 4.390, *p* = 0.003)

In this experiment, we tested the effects of acute BEA on EMG in response to CRD using two different stimulation parameters. Acute BEA with pain parameter (set 1; BEA-100Hz), but not motility parameter (set 2; BEA-25Hz), significantly reduced EMG in response to CRD in AA-treated male rats compared to the Sham stimulation (Fig 2). BEA dramatically reduced EMG at 20, 40, 60, and 80 mmHg [Sham vs. BEA (20 mmHg, 596.28±102.1 vs. 200.4±46.39, *p* = 0.027; 40 mmHg, 1115±108.89 vs. 520.8±74.97, *p* = 0.0002; 60 mmHg, 1371±128.38 vs. 760.9±87.69, *p* < 0.0001, 80mmHg, 1534±148.09 vs. 912.6±94.39, *p* < 0.0001), Bonferroni's multiple comparisons tests, Fig 2A], but not at 10 mmHg (Sham vs BEA, 254.3±76.80 vs 43.96±9.43, *p* = 0.619, Bonferroni's multiple comparisons tests, Fig 2A) compared to the Sham group. More importantly, BEA with motility parameter (BEA-25Hz) had no effects on visceral pain in AA-treated male rats (Two-way repeated measures ANOVA, *p* = 0.326, Fig 2B).

#### Acute SNS did not improve visceral pain

Next, we investigated whether direct sacral nerve stimulation (SNS) ameliorates visceral pain in AA-treated male rats. The effect of SNS with the same pain parameters was less potent, and there were no significant differences in EMG in response to CRD between the Sham and the SNS group (Two-way repeated measures ANOVA, *p* = 0.578, Fig 2C).

#### TEA via surface electrodes improved visceral pain

Previous studies in humans suggest that TEA at the acupoints of ST36 improved visceral pain in patients with IBS (Huang et al. 2022; Hu et al. 2022). Moreover, TEA has several advantages, including its non-invasiveness and home-based therapy. Therefore, we tested whether TEA using surface electrodes had a similar ameliorating effect on visceral pain in AA-treated male rats. We found that acute TEA improved visceral pain in AA-treated male rats (Fig 2D). TEA decreased EMG responses, in comparison with the Sham group, during CRD at 40, 60, and 80 mmHg [Sham vs. TEA (40 mmHg, 1189±94.16 vs. 550.0%±86.84, *p* = 0.001; 60mmHg, 1475±121.82 vs. 959.4±149.73, *p* = 0.012; 80 mmHg, 1729±171.63 vs. 1124±118.40, *p* = 0.002), Bonferroni's multiple comparisons tests, Fig 2D], but not at 10 and 20 mmHg [Sham vs TEA (10 mmHg, 133.1±40.42 vs 116.5±37.95, *p* > 0.99; 20 mmHg, 457.1±118.36 vs 259.8±81.92, *p* > 0.99), Bonferroni's multiple comparisons tests].

#### Acute UEA improved visceral pain

**Fig 3:**
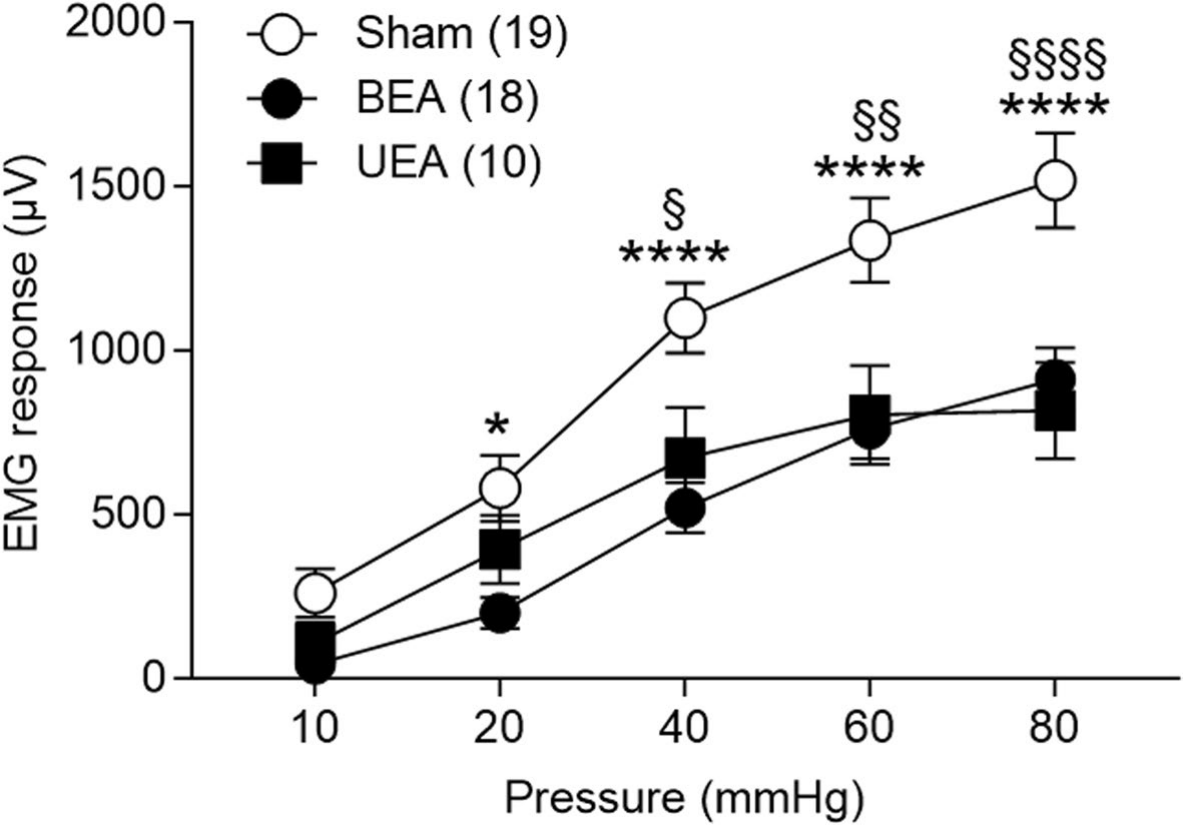
UEA improved visceral pain in comparison with BEA in AA-treated male rats. UEA decreased EMG responses to CRD compared to the Sham group in AA-treated male rats (Two-way repeated measures ANOVA, *F* (8, 176) = 3.62, *p* = 0.0006)

Clinically, unilateral stimulation is more straightforward to implement as it allows the subject to resume regular activities. Accordingly, we tested whether UEA had a similar beneficial effect on visceral pain as BEA. In AA-treated male rats, we observed that UEA showed a similar beneficial effect on visceral pain as BEA (Fig 3). UEA decreased EMG in response to CRD at 40, 60, and 80 mmHg [Sham vs. UEA (40 mmHg, 1101±104.13 vs. 671.9±146.07, *p* = 0.022; 60 mmHg, 1345±124.28 vs. 804.3±141.98, *p* = 0.002, 1506±143.0 vs. 817.8±139.14, *p* < 0.0001), Tukey's multiple comparisons tests, Fig 3], but not at 10 and 20 mmHg [Sham vs UEA (10 mmHg, 258.0±72.85 vs 107.3±61.71, *p* = 0.62; 20 mmHg, 585.4±97.30 vs 394.2±98.13, *p* = 0.46), Tukey's multiple comparisons tests, Fig 3]. No difference was noted between UEA and BEA.

#### Acute BEA improves visceral pain in female rats

**Fig 4:**
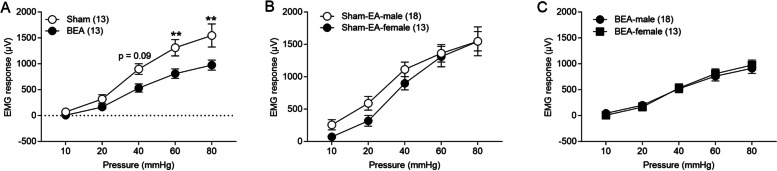
Acute BEA improves visceral pain in AA-treated female rats. **A** BEA decreased EMG responses compared to the Sham group (Two-way repeated measures ANOVA, *F* (4, 96) = 3.274, *p* = 0.014). There were no significant differences between (**B**) Sham treatment (Two-way repeated measures ANOVA, *F* (4, 116) = 0.865, *p* = 0.487) and (**C**) BEA treatment (Two-way repeated measures ANOVA, *F* (4, 116) = 0.389, *p* = 0.815) in both male and female rats

IBS is more commonly diagnosed in women than in men. Studies have shown that women are about two to three times (Kim and Kim 2018) more likely to be diagnosed with IBS. Therefore, we tested whether acute BEA was effective in AA-treated female rats. Interestingly, BEA in female rats demonstrated similar ameliorating effects in reducing visceral pain. BEA-100Hz in female rats improved EMG in response to CRD at 60 and 80 mmHg [Sham vs BEA (60 mmHg, 1311±150.92 vs 810.2±87.44, *p* = 0.007; 80 mmHg, 1547±214.39 vs 975.7±92.98, *p* = 0.001), Bonferroni's multiple comparisons tests, Fig 4A]. We did not observe any significant differences at 10, 20, and 40 mmHg [Sham vs BEA (10 mmHg, 72.5±35.10 vs 7.43±19.93, *p* > 0.999; 20 mmHg, 319.6±79.32 vs 166.4±36.59, *p* > 0.999; 40 mmHg, 899.3±98.32 vs 533.3±74.39, *p* = 0.095), Bonferroni's multiple comparisons tests, Fig 4A]. More importantly, Sham-EA and BEA had similar effects on EMG responses to CRD in AA-treated male and female rats [Sham-EA (male vs female, Two-way repeated measures ANOVA, *p* = 0.487, Fig 4B] and [BEA (male vs female, Two-way repeated measures ANOVA, *p* = 0.815, Fig 4C].

## Discussion

Our results demonstrate that acute BEA at ST36 acupoints improved visceral pain in AA-treated IBS rats. This finding is consistent with our previous study, where EA at ST36 acupoint with a similar stimulation protocol significantly reduced visceral hypersensitivity in rats (Chen et al. 2021a). Moreover, acute applications of UEA and TEA at ST36 demonstrated effectiveness in alleviating visceral pain in IBS rats. These results suggest that TEA is as effective as direct ST36 stimulation (BEA). However, acute SNS stimulation did not reduce visceral pain in AA-treated rats. Previous studies demonstrated that acute SNS at 14 Hz, pulse width of 330 ms, and stimulation amplitude of 40% MT normalized acute restraint stress-induced visceral hypersensitivity in rats (Jiang et al. 2019). Other research demonstrated that SNS with the 5 Hz, 500 μs, 10 seconds on, 90 seconds off parameters increased vagal activity and decreased sympathetic activity in 2, 4, 6-Trinitrobenzenesulfonic acid (TNBS) induced rats (Tu et al. 2020a; Zhang et al. 2020). The variation in results could be attributed to differences in the animal model and stimulation parameters used in these studies. Furthermore, our results demonstrate that acute BEA at ST36 improved visceral pain in female rats, suggesting similar efficacy between the sexes. However, it is important to note that the estrous cycle was not monitored in our study; therefore, the influence of the cycle phase could not be excluded.

The animal model we used in this study to induce IBS is well-established. These animals develop visceral hypersensitivity in adulthood, and the visceral pain response can be measured with EMG in response to CRD (Xu et al. 2008; Xu et al. 2009). CRD is a reproducible and reliable visceral stimulus, which is helpful in assessing visceral pain (Ness and Gebhart 1988). The abdominal EMG is a well-established method for assessing visceral pain in animal models that measure the electromyogram signal (reflecting abdominal muscle contractions) generated during the CRD. Acupoint ST36 stimulation is most commonly used in clinical settings to treat GI disorders, including IBS (Moon et al. 2023). Furthermore, acupoint ST36 is a critical site that modulates sympathetic and parasympathetic nervous systems since it is in the vicinity of peroneal, sciatic, and tibial nerves. Stimulation at ST36 impacts distal gut functions through anatomical proximity and influences upper gut functions through a functional connection with the central and vagal nerve systems (Liu et al. 2021; Lu et al. 2019; Ma et al. 2014). Accordingly, we chose to use AA-treated IBS model rats, EMG as a surrogate for pain measurement, and ST36 as the focus of our research study.

Most interestingly, the findings of this comparative methodological study demonstrated similar ameliorating effects between unilateral and bilateral stimulation, and between direct ST36 stimulation and transcutaneous ST36 stimulation. These findings suggest a novel therapeutic approach for pain in IBS: unilateral transcutaneous ST36 stimulation. This unilateral TEA method will have several advantages: (1) it is completely noninvasive; (2) it can be self-administered at home since it does not use needles: (3) the unilateral stimulation (preferably use of a wireless wearable stimulator) does not interfere with daily activity of the user.

IBS is more predominant in women than in men, with a female-to-male ratio of 2–2.5:1 (Kim and Kim 2018). However, its pathophysiologic mechanisms are still unclear. While both men and women with IBS experience similar symptoms, including abdominal pain or discomfort, diarrhea, and constipation, women experience more abdominal pain and constipation-related symptoms. Sex hormones are thought to play a critical role that most influences the clinical manifestation and physiologic responses in men and women with IBS. Some research suggests that women may have increased sensitivity to visceral pain compared to men. This heightened sensitivity could contribute to differences in the perception and experience of IBS symptoms between genders (Kim and Kim 2018; Chial and Camilleri 2002). While IBS can significantly impact the quality of life for both men and women, studies have found that women with IBS may experience more severe symptoms and more significant impairment in quality of life compared to men. Understanding these similarities and differences in the medical care environment and applying them to IBS patients can help healthcare providers tailor treatment approaches for individuals with IBS.

Alteration in the inputs from the gut, known as afferent sensitization, is thought to play a crucial role in pain sensitization in patients with IBS (Mayer et al. 2023; Midenfjord et al. 2021). Under pathophysiological conditions, primary visceral afferent neurons, aka vagal afferent, convey pain signals from the viscera to the NTS (Gebhart 2000). On the other hand, spinal visceral afferent neurons from the intestinal tract are located in different spinal segments, and this viscerosomatic cross-organ sensitization may be involved in a central mechanism of nociceptive signaling. For example, increased expression of transient receptor potential vanilloid type-1 (TRPV1) contributes to visceral hypersensitivity and pain (Perna 2021). Thus, afferent sensitization is an important factor contributing to pain in IBS. EA at ST36 significantly decreased chronic visceral hypersensitivity and colon 5-HT3 receptor levels in AA-treated rats (Chu et al. 2011). Moreover, EA decreased rectal sensitivity by decreasing TRPV1 in both colon and dorsal root ganglions (Chen et al. 2021a). Pre-EA at acupoint EX-B2 significantly reduced intracolonic formalin-induced visceral pain by decreasing p38 phosphorylation and c-Fos expression in the spinal cord and colon (Xu et al. 2010). Colonic biopsies from IBS patients had elevated mucosal N-methyl-D-aspartate receptor (NMDAR) levels that were positively correlated with the severity and rate of recurrence of abdominal symptoms (Qi et al. 2017). Clinical and animal studies demonstrated that administering NMDAR antagonist dextromethorphan in IBS patients and MK801 in mice blocked somatic and visceral hypersensitivity (Qi et al. 2017; Zhou et al. 2011). Moreover, the injection of D-2-amino-5-phosphonopentanoate (AP5) into the rostral ventromedial medulla (RVM) inhibited visceral pain (Sanoja et al. 2010), and locus coeruleus-RVM circuit was found to be essential for the comorbidity of colorectal visceral pain (Kong et al. 2023). EA at ST36 and ST37 improved visceral hyperalgesia, decreased c-Fos, and NMDAR expression in the RVM in IBS model rats (Qi and Li 2012), suggesting an analgesic effect of EA, which may mediated by inhibiting NMDAR activation in the RVM. These studies have suggested that EA desensitizes visceral and sensory afferents and improves visceral pain in IBS.

Chronic, low-grade inflammation is thought to play a critical role in the pathophysiology of IBS (Bercik et al. 2005; El-Salhy et al. 2013). Increased levels of inflammatory cytokines, including interleukin (IL)-6, IL-1β, IL-8, and tumor necrosis factor (TNF)-α, have been reported in the blood and serum of IBS model animals and IBS patients (Dinan et al. 2006; Liebregts et al. 2007; Heel et al. 2002). A clinical study demonstrated that increased levels of serotonin (5-HT) in IBS patients contributed to abdominal pain (Cremon et al. 2011). Thus, low-grade inflammation may contribute to pain in IBS patients. EA reduced pro-inflammatory cytokines, including TNF-α, IL-1β, and IL-6, and suppressed myeloperoxidase activity in the colon via the autonomic mechanism (Jin et al. 2019). Another research showed that EA suppressed the expression of inflammatory cytokines, such as IL-8, IFN-γ, and TNF-α and in water avoidance stress (WAS) induced IBS mice and alleviated pain by suppressing the expression of inflammatory cytokines, such as IL-8, IFN-γ, and TNF-α (Mengzhu et al. 2023). Results from these studies suggest that EA may improve pain in IBS by reducing inflammation.

The role of the cholinergic anti-inflammatory pathway in reducing inflammation in GI disorders is well-documented (Borovikova et al. 2000; Ghia et al. 2006; Goverse et al. 2016). This pathway functions through vagal efferent fibers that link to enteric neurons and release acetylcholine (Pavlov et al. 2003; Tracey 2002). Disruption of this pathway can synthesize pro-inflammatory cytokines, including TNF- α and IL-1, which may lead to intestinal mucosal inflammation, thus contributing to visceral pain. Upon parasympathetic activation, enteric neurons release acetylcholine, which interacts with α7 nicotinic acetylcholine receptors (α7nAChRs) on macrophages, inhibiting pro-inflammatory cytokine production (Cheng et al. 2020). Moreover, by activating parasympathetic outflow, the cholinergic anti-inflammatory pathway inhibits macrophage activation and regulates inflammation (Borovikova et al. 2000). EA at ST36 restored the impaired colonic contraction and transit induced by rectal distension by enhancing vagal activity and mediated via the cholinergic pathway (Jin et al. 2015).

The gastrointestinal epithelium acts as a barrier, preventing the penetration of harmful substances in the lumen from other tissues via the intestinal mucosa. Human and animal studies have reported increased intestinal permeability in GI disorders (Camilleri 2012; Coeffier et al. 2010). Previous studies reported that alteration in epithelial tight junctions (TJ) proteins, such as Zonula Occludens (ZO-1), Claudins, and Occludin, led to epithelial barrier dysfunction and contributed to the pathogenesis of IBS and pain (Coeffier et al. 2010; Martinez et al. 2012; Nusrat et al. 2000). EA increased ZO-1 and enhanced the repair of the intestinal mucosal barrier by decreasing corticotropin-releasing factor-receptor 1 expression in the gastrointestinal mucosa (Chen et al. 2019), as well as EA improved intestinal permeability by increasing the expression of TJ proteins in IBS mice and rats (Mengzhu et al. 2023; Li et al. 2022). Thus, EA may modulate TJ, improving mucosal barrier function and ameliorating visceral hypersensitivity and pain.

Mast cells are widely distributed in the colonic mucosa and release substances like histamine, proteases, growth factors, prostaglandins, and cytokines. These mediators were reported to increase the excitability of enteric (Reed et al. 2003) and primary afferent neurons (Nozdrachev et al. 1999), leading to visceral hypersensitivity (Gebhart 2000). Previous studies suggested that mast cell activation correlated with the severity of abdominal pain (Cremon et al. 2011; Barbara et al. 2004). Furthermore, mast cell dysfunction compromises epithelial barrier function, which alters mucosal permeability, potentially leading to altered bowel function and pain (Hasler et al. 2022). A clinical study demonstrated that the number and activity of mucosal mast cells in IBS patients positively correlated with the degree of intestinal permeability (Lee et al. 2013). Thus, from these preclinical and human studies, it is clear that mast cells are more likely to be activated in patients with IBS, releasing mediators known to interact with nerve endings and trigger pain. A recent research study reported that the EA at ST36 acupoint ameliorates post-inflammation rectal hypersensitivity by down-regulating mast cells activated nerve growth factor and tropomyosin receptor kinase A (Chen et al. 2021a).

Whole-brain imaging techniques such as functional magnetic resonance imaging (fMRI) (Ma et al. 2020; Zhao et al. 2021) have been used to assess the mechanism of pain and suggested that changes in brain structure and functional connections (FCs) correlate with pain in IBS patients. Alteration of the serotonergic signaling in the emotional arousal circuit has been reported in both male and female IBS patients, which contributes to visceral hypersensitivity (Hubbard et al. 2015). Moreover, IBS patients had alterations in grey matter in brain areas associated with cognitive and evaluative functions (Seminowicz 2010; Zhao et al. 2023). Abnormal FCs in brain areas, including the hippocampus, occipital gyrus, and cerebellum, have been reported in IBS patients, and acupuncture treatment has improved these FCs (Ma et al. 2020; Chu et al. 2012). EA may exert an analgesic effect in IBS by enhancing the FC between the hippocampus and various brain regions and modulating the default mode and sensorimotor networks (Zhao et al. 2021). Thus, EA alleviates visceral pain in IBS model rats by regulating the peripheral, central, and endocrine systems, reducing inflammation, improving colon permeability, stabilizing mast cell function, and altering brain activity.

In this project, we chose to stimulate acupoint ST36 and compare it with direct stimulation of the sacral nerve because of the following: ST36 is in the vicinity of peroneal, sciatic and tibial nerves; these nerves converge to the sacral nerve. Accordingly, neuroanatomically, electrical stimulation at ST36 and the sacral nerve might have similar effects. However, ST36 electrical stimulation can be accomplished noninvasively, using the method of TEA. Although not investigated in this project, previous studies have suggested two possible pathways: 1) direct efferent stimulation, i.e., ST36 EA and SNS directly activate the sacral efferent; 2) afferent stimulation: both ST36 stimulation and SNS have been reported to activate the nucleus tractus solitarius (NTS), resulting in activation of the vagal efferent (Iwa et al. 2007; Tu et al. 2020b).

In conclusion, transcutaneous ST36 stimulation is as effective as direct ST36 stimulation and unilateral ST36 stimulation is comparable to bilateral stimulation. Development of a novel therapy using unilateral transcutaneous ST36 stimulation is warranted.

## Data Availability

All data and materials are available upon request to the corresponding authors.
